# Morphometrics and Structural Changes of “Terung Asam” Sarawak (*Solanum lasiocarpum* Dunal) During Growth and Development

**DOI:** 10.21315/tlsr2023.34.3.2

**Published:** 2023-09-30

**Authors:** Albert Ting Koon Soon, Phebe Ding, Shiamala Devi Ramaiya

**Affiliations:** 1 Department of Crop Science, Faculty of Agriculture, Universiti Putra Malaysia, 43400 Universiti Putra Malaysia Serdang, Selangor, Malaysia; 2 Department of Crop Science, Faculty of Agricultural and Forestry Sciences, Universiti Putra Malaysia Bintulu Sarawak Campus, Nyabau Road, 97008 Bintulu, Sarawak, Malaysia

**Keywords:** Cellular Structure, Exocarp, Single Sigmoid Curve, Trichomes, Struktur Sel, Eksokarp, Keluk Sigmoid Tunggal, Trikoma

## Abstract

“Terung asam” Sarawak (*Solanum lasiocarpum* Dunal) is an underutilised fruit vegetable. Information on the fruit growth is very lacking. Thus, this study was conducted to characterise fruit growth pattern based on physical characters and cellular structures. Data were recorded weekly from fruit set until senescence. All the morphological growth of “terung asam” fruit exhibits a single sigmoid growth pattern that fitted well to logistic model. There are three distinct phases of growth, i.e., S1, S2 and S3. At S1, the size of fruit cells was small without intercellular spaces. As fruit grew to S2, cell size increased with distinct vascular tissues. By S3, fruit has achieved its maximum size with green peel turn to yellow and finally golden yellow at late S3. Cuticle and two types of trichomes formed the outer layer of fruit. The thickness of fruit exocarp increased while density of trichomes decreased as fruit developed.

## Abstrak

Terung asam Sarawak (*Solanum lasiocarpum* Dunal) merupakan sayur buah yang jarang digunakan. Maklumat tentang pertumbuhan buah ini adalah sangat kurang. Justeru, kajian ini dijalankan untuk mencirikan corak pertumbuhan buah berdasarkan ciri fizikal dan struktur sel. Data direkodkan setiap minggu bermula dari proses pembentukan buah sehingga proses penuaan. Semua pertumbuhan morfologi buah terung asam mempamerkan corak pertumbuhan sigmoid tunggal yang berpadanan dengan model logistik. Terdapat tiga fasa pertumbuhan yang nyata, iaitu S1, S2 dan S3. Pada S1, saiz sel buah adalah kecil tanpa ruang antara sel. Apabila buah berkembang menjadi S2, saiz sel bertambah manakala tisu vaskular jelas kelihatan. Menjelang S3, buah telah mencapai saiz maksimum di mana kulit hijau bertukar kepada kuning dan akhirnya kuning keemasan pada penghujung S3. Kutikular dan dua jenis trikoma membentuk lapisan luar buah. Ketebalan eksokarp buah meningkat manakala ketumpatan trikoma berkurangan apabila buah berkembang.

**Highlights**

- “Terung asam” Sarawak fruit exhibits a single sigmoid growth pattern that fitted well to logistic model.
- Three phases of growth, i.e., S1, S2 and S3 were identified in “terung asam” Sarawak fruit with total of 16 weeks from flower anthesis to fruit senescence.
- Cellular structures of “terung asam” Sarawak fruit change with its growth and development.

## INTRODUCTION

“Terung asam” Sarawak, or *Solanum lasiocarpum* from the Solanaceae family, is a round-sourish fruit vegetable. The colour of the fruit changes from green to golden yellow as the fruit ripens (Ting & Ding 2021). It is indigenous to Sarawak, Malaysia, and popular among local communities where it is served in their daily dishes or used in food processing. The unique sour taste of “terung asam” fruit is often used to substitute tamarind in *asam pedas* (spicy fish dish) for the local and making Sarawak *asam pedas* as a special dish among all *asam pedas* recipes. The commercially available processed “terung asam” Sarawak products are ice cream, jam, juice, cordial drink, dehydrate fruit slices, rolls, Tiung Chips cookies, cracker, jelly, cake, sambal and even as preserved food in syrup and brine solution (Shariah *et al*. 2013). Thus, the demand for this fruit vegetable in Sarawak is up surging, and it is highly priced at USD1.43–USD2.39 per kg, depending on its size and quality. The “terung asam” fruit price is on par with the price of other fruit vegetables in Sarawak such as tomato (USD2.06) and eggplant (USD2.11) (Federal Agriculture Marketing Authority [FAMA] 2021).

Fruit growth, which refers to a change in the fruit’s physical measurements, such as weight, length, width, and volume, is of great importance as an indicator to determine fruit size and quality (Mohammad & Ding 2019). The growth could partly due to macro (physical aspects) and micro (cellular aspects) changes after successful pollination and fertilisation of a flower. Cellular structure studies have been carried out to understand and justify some of the physical changes in fruit size and volume. These changes are closely associated with the chronological development of a fruit, starting from flowering to fruit maturation and, finally, senescence (Jamaludin *et al*. 2011; Mohammad & Ding 2019; Mijin & Ding 2020; Tee *et al*. 2011). The obtained cumulative data of the increment in physical measurements can then be used to show a simple- or double-sigmoid growth curve, depending on fruit cultivars which was due to the cooperation between the velocity and duration of fruit enlargement (Pei *et al*. 2020).

The growth curve of a fruit is due to developing of tissue types at their own rate and in accordance with their own programme (Boini *et al*. 2019). There are three developmental stages in a fruit growth curve: cell division, cell expansion, and maturation (Jamaludin *et al*. 2011; Mijin & Ding 2020; Mohammad & Ding 2019; Tee *et a*l. 2011). Fruit ripening occurs at the end of fruit maturation which turn fruit to palatable food for consumption. The fruit growth curve is able to provide duration needs for each developmental stages of a fruit so that proper management can be taken to enhance the fruit quality while fruit is still attaching to the mother plants. The duration for a fruit to complete its growth and development or fruit life cycle differs in each species. For example, *Carissa congesta*, a kind of underutilised small berry fruit weighing about 3.5 g per fruit, takes 13 weeks after anthesis to ripen (Mohammad & Ding 2019). Contrary, red-fleshed dragon fruit (*Hylocereus polyrhizus*) which also a kind of berry fruit weighing about 600 g per fruit, takes 5 weeks after anthesis to ripen (Jamaludin *et al*. 2011). It seems fruit size and life cycle does not have any correlation.

Although “terung asam” Sarawak is a popular and common fruit vegetable in Sarawak, scientific research, especially on fruit growth and development is almost nil. Thus, a study was carried out to characterise its growth patterns based on physical characters while fruit cellular structures during growth and development were also elucidated. Information on the changes in structural aspects during fruit growth and development is able to provide important insight of a fruit behaviour that would affect its handling especially after harvesting. Hence, it is essential to determine cellular structure of “terung asam” Sarawak too for ensuring quality fruit production.

## MATERIALS AND METHODS

### Plant Materials

The study was conducted using 30 healthy *Solanum lasiocarpum* plants grown in pots (diameter 50 cm; height 43 cm) at Field 15 (2°59′06.1″ N 101°44′10.5″ E), Universiti Putra Malaysia. In total, 230 fully bloomed flowers of “terung asam” were randomly tagged. A week after anthesis (WAA), flowers that set fruit were recorded as 1 WAA. To minimise experimental errors due to crop load, the thinning process was carried out to allow only one well-developed fruit per cluster (Ding & Mashah 2016). Then, 5 to 10 “terung asam” fruits were harvested weekly from 8 a.m. to 10 a.m. in the morning from the 30 plants. The harvested fruit was immediately sent to the Laboratory of Postharvest, Faculty of Agriculture, Universiti Putra Malaysia, where the fruit were randomly distributed for respective assessments.

### Fruit General Observation and Growth Characteristics

The growth and development of “terung asam” Sarawak flowers to fruitlets were recorded using a digital camera as field observation. A non-destructive sampling method was used for fruit length and diameter, where the fruit is still attached to the plants. A total of 12 fruits were tagged and selected from 12 plants for fruit polar length and equatorial diameter determination. Measurements were conducted using the same fruit from 1 WAA until senesce (fell from trees) with the help of a digital Vernier calliper (Model CD-8” CSX, Mitutoyo, Japan) and expressed in centimetres. Fruit polar length was measured from the stem end to the proximal end of each fruit. Fruit equatorial diameter was taken from the widest measurement of the fruit at the equatorial region. Mean values of fruit length and diameter were calculated. Freshly harvested fruit was used for fresh weight and volume determination. Fresh weight was determined using an electronic balance (Model B303-S, Mettler Toledo, Greifensee, Switzerland). To obtain accurate readings, the balance was set, placed, and levelled properly, and readings were expressed in gram. The volume of fruit was estimated by immersing the fruit in a water-filled measuring cylinder (500 mL), where the volume of water displaced by complete immersion was recorded (Mohammad & Ding 2019).

### Cellular Structure Studies

For light microscopy (LM) and scanning electron microscopy (SEM) studies, the method was carried out according to Mohammad and Ding (2019), with some modifications on the sample processing periods and equipment used. A tissue size of 1 cm^3^ was taken from the fruit equator and immediately fixed in fixative-filled glass bottles (200 mL) containing 37% formaldehyde: 100% acetic acid: 95% ethanol at a 0.5:0.5:9 ratio. Samples were left in a fixative state for at least seven days before being further processed. The fixed tissues were vacuumed for 24 h to remove the air bubbles in them. Tissues were then dehydrated for 30 min each in an increasing gradient of ethanol series, i.e., 30%, 50%, 75%, 90%, 95% and 100% (two times) before being kept in methyl benzoate for 24 h. The tissues were infiltrated with isopropanol and paraffin (melting point 65°C) in ratios of 1:1, 1:3, and 100% of paraffin (3 h for each ratio) before being infiltrated with 100% paraffin for 24 h. Then, individual tissue was embedded in a paper box filled with 100% paraffin, and the block was left overnight at room temperature, allowing it to be solidified. Solidified blocks were sectioned (thickness of 12 μm) using a sliding microtome (Model SM 2000 R, Leica, Wetzlar, Germany). Waxy strips containing sections of samples were placed on the surface of warm water (50°C) before being transferred onto slides. After drying in an oven for 2 h, the sections were immersed in a xylene solution for a total of 15 min (3 times for 5 min) for dewaxing purposes. Then, the sections were rehydrated where they were immersed in a decreasing gradient of ethanol series, i.e., 100%, 95%, 90%, 75%, 50% and 30% for 5 min each concentration before being immersed in distilled water for 5 min. The sections were stained with 0.05% safranin O and mounted using dibutylphthalate polystyrene xylene after the slides were dried. The slides were viewed under a compound microscope (MT 5300H, Meiji Techno Co., Ltd., Saitama, Japan) equipped with a digital camera (Model OM-D E-M10 Mark II, Olympus, Japan).

For the SEM study, the fixed tissues were vacuumed for 24 h before being rinsed in deionised water for 10 s and then post-fixed in 1% osmium tetroxide for 2 h. The tissues were rinsed again in deionised water for 10 s before being subjected to a dehydration process in an increasing gradient of acetone series (30%, 50%, 70%, 80%, 90% and 100%) for 30 min in each concentration, with another dehydration in 100% acetone for 1 h. Samples were dried using a critical point drier (Balzer CPD 030, Wetzlar, Germany), mounted on metal stubs, and sputter-coated (Sputter Coater JEOL JFC-1600, Tokyo, Japan) in gold for 2 min. Finally, the samples were viewed under the high vacuum of SEM (JOEL JSM-5610LV, Tokyo, Japan) at an acceleration voltage of 15 kV, with a working distance of 39 mm. Fruit exocarp thickness from each growth stage was determined using the ImageJ version 1.46 computer program developed by Wayne Rasband, and readings were recorded in μm and analysed.

### Statistical Analysis

The experiment was laid out in a completely randomised design, with 12 fruit replications for each WAA. The morphological traits of fruit growth were subjected to logistic regression analysis fitting to equation Y = α/(1 + βe^(−δx)^), where Y is the trait analysed (length, diameter, fresh weight or volume), coefficient α is the maximum size attained by the fruit, β controls the rate of growth, δ is the effect of the slope of the growth curve, and x is time (in WAA). The rest of the parameters were subjected to analysis of variance (ANOVA) using Statistical Analysis System (SAS 9.4). When the F-values of ANOVA showed statistical significance (*p* ≤ 0.05), the mean values were further separated using Duncan’s multiple range test (DMRT).

## RESULTS

### Fruit Growth

**Figure 1:**
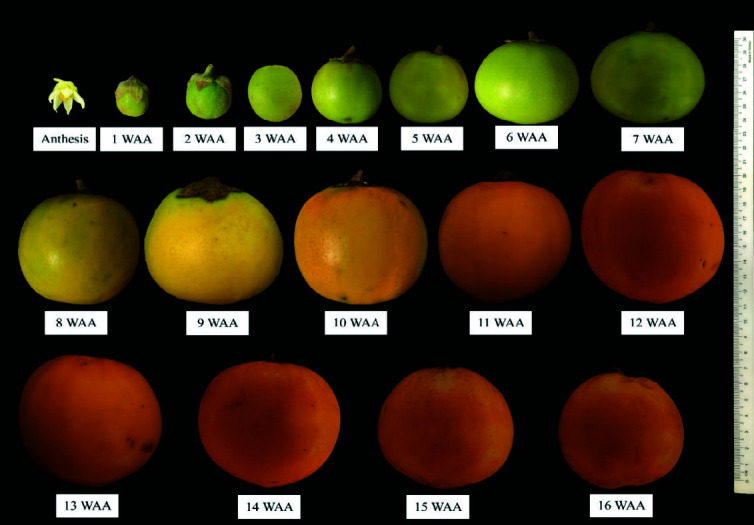
Photograph chart of “terung asam” Sarawak fruit, from anthesis (full bloom flower) until the end of fruit development at 16 weeks after anthesis (WAA).

**Figure 2:**
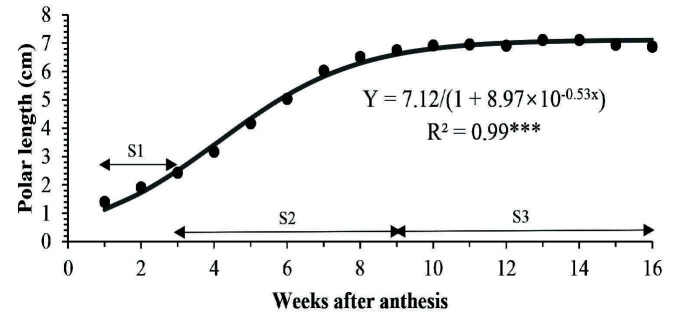
Polar length of “terung asam” fruit as the weeks after anthesis (WAA) progressed. *Note*: The stages were as follows: S1, from 1 until 3 WAA; S2, from 4 until 9 WAA; S3, from 10 until 16 WAA. The solid line indicates the fitted line of the logistic model. (*n* = 12 fruits). Significant at ***p ≤ 0.001.

**Figure 3:**
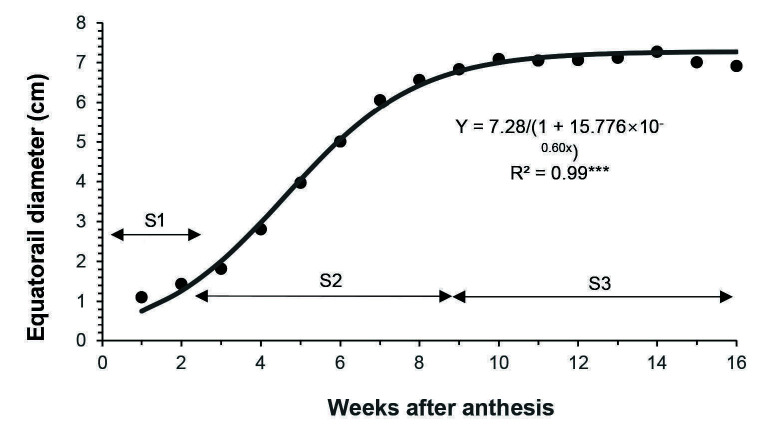
Equatorial diameter of “terung asam” fruit as the weeks after anthesis (WAA) progressed. *Note*: The stages were as follows: S1, from 1 until 3 WAA; S2, from 4 until 9 WAA; S3, from 10 until 16 WAA. The solid line indicates the fitted line of the logistic model. (*n* = 12 fruits). Significant at ***p ≤ 0.001.

**Figure 4:**
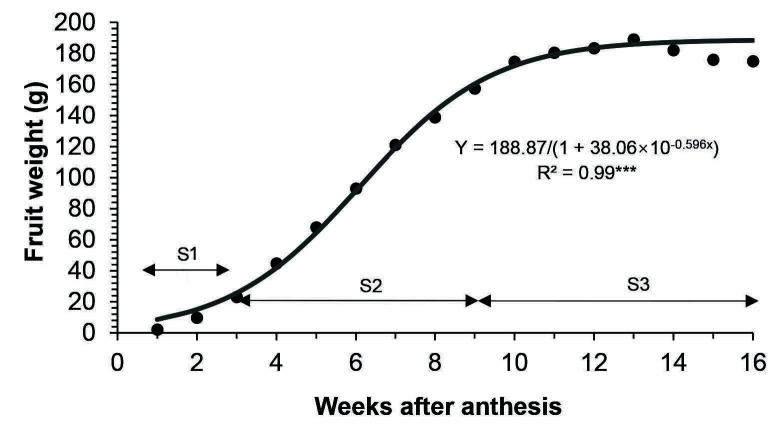
Fruit weight of “terung asam” fruit as the weeks after anthesis (WAA) progressed. *Note*: The stages were as follows: S1, from 1 until 3 WAA; S2, from 4 until 9 WAA; S3, from 10 until 16 WAA. The solid line indicates the fitted line of the logistic model. (*n* = 12 fruits). Significant at ***p ≤ 0.001.

**Figure 5:**
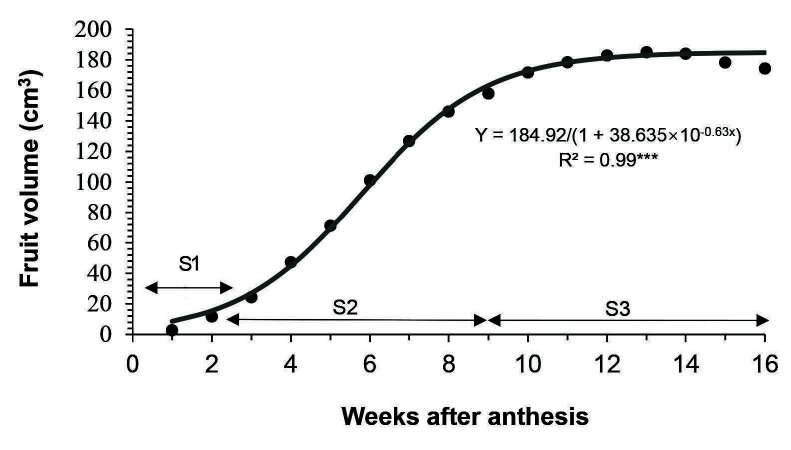
Fruit volume of “terung asam” fruit as the weeks after anthesis (WAA) progressed. *Note*: The stages were as follows: S1, from 1 until 3 WAA; S2, from 4 until 9 WAA; S3, from 10 until 16 WAA. The solid line indicates the fitted line of the logistic model. (*n* = 12 fruits). Significant at ***p ≤ 0.001.

“Terung asam” Sarawak fruit took 16 weeks after anthesis (WAA) to develop from the anthesis of a flower to senescence that led to falling of fruit from the plants (Fig. 1). The fruit increased in size and changed in peel colour from green to yellow, then golden yellow at the end of its life cycle. The polar length, equatorial diameter, fresh weight and volume of fruit morphology throughout 16 weeks of development are shown in Figs. 2, 3, 4 and 5, respectively. These morphological traits fitted well to the logistic model, each with an estimated intercept and quadratic regression coefficient of 0.99 (Table 1).

Generally, length, diameter, fresh weight and volume of “terung asam” fruit increased with growth and displayed a single sigmoid growth curve. The growth occurred in three prominent phases, namely S1, S2 and S3. The velocity of fruit growth was relatively slow at the S1 phase, where the fruit length, diameter, weight and volume increased by 73.06%, 64.59%, 198.14% and 215.29%, respectively during the first three weeks of development. By S2, the growth increased exponentially after 3 WAA and halted at 9 WAA. At this point, length, diameter, weight and volume increased by 112.83%, 143.35%, 656.59% and 605.15%, respectively. When the fruit entered 10 WAA, the growth became constant and remarked with the S3 phase, which lasted 7 weeks before the fruit detached from the mother plants due to senescence.

### Cellular Structural Changes

**Figure 6:**
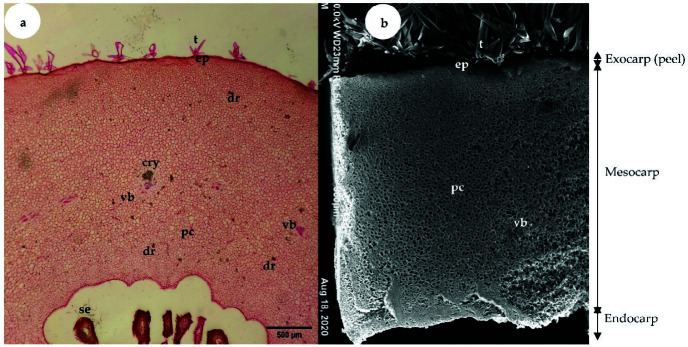
(a) LM micrograph shows a transverse section of “terung asam” fruit at 2 weeks after anthesis (S1). ×40; bar = 500 μm. (b) Scanning electron microscopy micrograph shows a transverse section of “terung asam” fruit at S1. ×50; bar = 500 μm (cry: crystalline material; dr: druse crystal; ep: epidermal cell; pc: parenchyma cells; se: seed; t: trichome; vb: vascular bundle).

Fruit tissues at 2, 5, or 8 and 13 WAA were processed to observe the cellular structures of “terung asam” Sarawak fruit during the S1, S2 and S3 phases of growth, respectively. The ovary wall of successfully fertilised “terung asam” flowers developed into a fruit pericarp with three distinct regions: exocarp, mesocarp and endocarp (Fig. 6). The exocarp composed by a layer of cuticle that covering the epidermal cells of the fruit. At S1, the exocarp of 2 WAA fruit was only 3.92 μm thick (Table 2). As growth progressed, the thickness of the exocarp of S2 fruit increased drastically by almost 15-fold and reached a thickness of 62.39 μm. From S2 to S3, the thickness of the exocarp increased by 26.69%.

**Figure 7:**
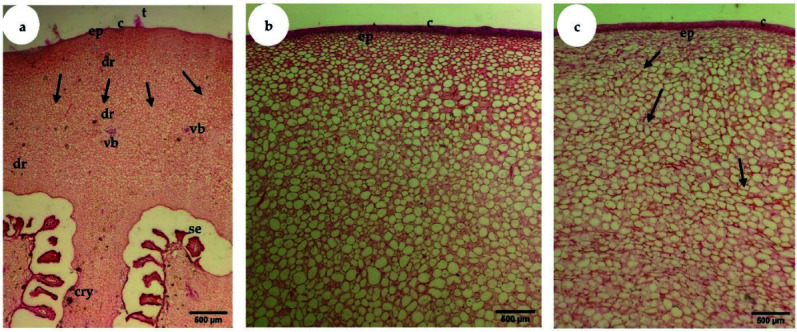
LM micrographs show transverse sections of “terung asam” fruit throughout the three phases (S1–S3) of growth and development. (a) Cellular structure at the S1 growth stage (fruit of 2 weeks after anthesis), where the cells actively divide in all directions (arrows). ×40; bar = 500 μm. (b) At the S2 or fruit of 8 weeks after anthesis, the size of the parenchyma cells in the mesocarp region was bigger and spherical compared to those in S1. ×40; bar = 500 μm. (c) By the S3 by using fruit of 16 weeks after anthesis, the parenchyma cells became bigger and irregular in shape (arrows) compared to the previous phase of cells. ×40; bar = 500 μm (c: cuticle; cry: crystalline material; dr: druse crystal; ep: epidermis; se: seed; t: trichome; vb: vascular bundle).

**Figure 8:**
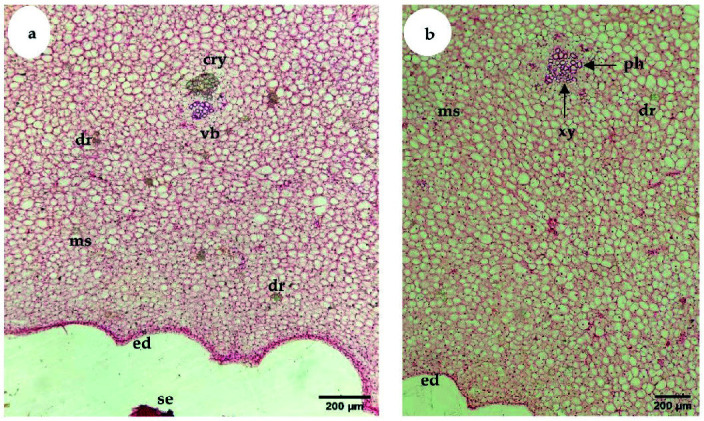
(a) LM micrograph shows a transverse section of “terung asam” fruit at the inner region of the mesocarp, next to the endocarp at S1. ×100; bar = 200 μm. (b) LM shows a transverse section of “terung asam” fruit at the inner region of the mesocarp, next to the endocarp at S2 (5 weeks after anthesis). ×100; bar = 200 μm (cry: crystalline material; dr: druse crystal; ms: mesocarp; ed: endocarp; ph: phloem; se: seed; vb: vascular bundle; xy: xylem).

The parenchymatous cells of the S1 phase fruit were small, irregular and tightly packed until intercellular spaces were hardly observed (Fig. 7a). Vascular bundles appeared as strand-like structures and were not clearly differentiated during S1. As fruit developed to the S2 phase, parenchymatous cells increased in size (Fig. 7b). As fruit developed to the last phase, the shape of the parenchymatous cells was found to be irregular and loosely packed with obvious intercellular spaces (Fig. 7c). In addition, vascular bundles with well-defined xylem and phloem tissues were not clearly seen in S1 (Fig. 8a), but the tissues were easily found in S2 (Fig. 8b).

**Figure 9:**
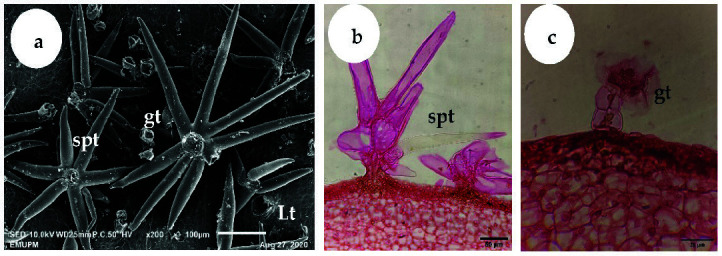
(a) Scanning electron microscopy micrograph shows a plan view of the “terung asam” fruit exocarp at S1. The young fruit exocarp is densely covered with both sessile porrect-stellate and glandular trichomes. ×200; bar = 100 μm. (b) LM micrograph shows a transverse section of “terung asam” fruit at S1. The exocarp of young fruit is densely covered with sessile porrect-stellate trichomes. ×400; bar = 50 μm. (c) LM micrograph shows a transverse section of “terung asam” fruit at S1. A glandular trichome is located at the exocarp of young fruit. ×1,000; bar = 20 μm (Lt: lenticel; spt: sessile porrect-stellate trichome; gt: glandular trichome).

The outermost exocarp or peel of “terung asam” fruit was covered by two types of trichomes, i.e., sessile porrect-stellate and glandular (Figs. 9a–9c). During the S1 phase, the outermost exocarp was densely covered by trichomes (Fig. 9a). As the fruit grew, the density of the trichomes reduced and hardly be found when the fruit ripened at S3 (Fig. 9c). In addition to trichomes, idioblast cells could also be found in fruit mesocarp (Figs. 7–8).

## DISCUSSION

“Terung asam” Sarawak fruit took 16 WAA or 112 days to develop into mature and ripe fruit, which eventually fell from the plant due to senescence (Figs. 2–5). The common fruit vegetable such as eggplants (*Solanum melongena* L.) takes about 60 days from fruit set to mature stage, whereas tomato fruit (*Lycopersicon esculentum)* takes about 40 to 70 days to grow and reach the “red ripe” stage (Srivastava & Handa 2005; Zaro *et al*. 2015). Compared to commonly cultivated species of Solanaceae family fruit, “terung asam” fruit had a much longer fruit cycle, which is most probably due to the “terung asam” plant has not gone through any selective breeding program. Cultivated eggplants are intensively selected for a few desired traits during domestication, including a short fruit cycle to increase their economic value (Meyer *et al*. 2012). The lack of breeding program for the “terung asam” plant also means that the research on this plant is very limited, and there is huge room to study this plant.

During 16 weeks of fruit development, “terung asam” fruit underwent three distinct growth stages, with extensive cell division occurring at the S1 phase (1 to 3 WAA). Fruits act as sink organs and are able to store photosynthetic products synthesized in leaves inside parenchyma cells in the form of sucrose or sorbitol (Fischer *et al*. 2012). The accumulation of sugar is vital in creating cellular water retention inside fruits. An increase in cellular sugar concentration will then cause water to flow into vacuoles that will exert pushing force toward the cytoplasm and cell walls, resulting in overall fruit enlargement. The role of fruit as a sink organ is obvious; “terung asam” fruit in the S2 phase grew rapidly. There was a presence of well-developed vascular bundles in S2 (Fig. 8b) compared to S1 (Fig. 8a), implying nutrients and water were being actively transported to growing tissues during fruit growth and development. This might explain the reason “terung asam” fruit attained exponential growth in the S2 phase, which can be clearly seen in the fruit’s morphological changes, i.e., length, diameter, weight, and volume (Figs. 2–5). The exponential growth at the S2 phase allowed the fruit to reach its maximum size by 9 WAA or a total of 6 weeks for the fruit to gain its final dimension.

After 9 WAA, there were no significant changes in fruit size, but the walls and membranes of cells had deteriorated when viewed under LM (Fig. 7c). As the fruit reached S3 phase, the shape of the parenchymatous cells was found to be irregular and loosely packed, with significant intercellular spaces (Fig. 7c). Some senescence-related physiological changes occur at ripening, causing membrane deterioration in a programmed cell death process (Bouzayen *et al*. 2010). In this process, ripening-related genes will code for cell wall degradation enzymes, which degrade the structure and reduce the strength of cell walls (Toivonen & Brummell 2008). This situation will lead to larger air spaces and reduce intercellular contact and firmness, indicating that the fruit has indeed ripened and become palatable. From the growth curve, it is clear that “terung asam” Sarawak takes 7 weeks to mature, ripen and finally senesce.

The findings of this study also illustrate that the thickness of the “terung asam” Sarawak fruit exocarp increased significantly as the fruit developed (Table 2). Fruit exocarp is crucial in regulating gas exchange, preventing massive water loss, filtering potentially damaging UV light, limiting invasion by pathogens, and protecting against mechanical injuries (Yeats & Rose 2013). These functions are essential throughout fruit growth and development since they have a high impact on fruit physiology, quality, and postharvest shelf life (Lara *et al*. 2014). However, the thick exocarp may give unpleasant mouthfeel of the fruit. The presence of trichomes and idioblast cells in the fruit may have also played an important role in providing protection against herbivore, and biotic and abiotic stresses. This may explain the plant could easily survive in the wild.

## CONCLUSION

“Terung asam” Sarawak fruit takes 16 weeks from flower anthesis to senescence. As the fruit develops, it exhibits a single sigmoid growth curve and displays three noticeable phases of growth, i.e., S1, S2 and S3 that takes 3, 6 and 7 weeks, respectively. From the morphological (weight, polar length, equatorial diameter and volume) changes, each phase of growth characterised by different growth rate. The thick fruit exocarp with trichomes could provide natural protection to the fruit and cause it resistant to pest and disease attack.

## Figures and Tables

**Table 1:** Logistic models fitted for length, diameter, fresh weight and volume against 1 to 16 weeks after anthesis of “terung asam” fruit according to R2.

Dependent variable, Y	Logistic model	R^2^
Length (cm)	Y = 7.12/(1 + 8.97 × 10^−0.53x^)	0.99
Diameter (cm)	Y = 7.28/(1 + 15.77 × 10^−0.60x^)	0.99
Fresh weight (g)	Y = 188.87/(1 + 38.06 × 10^−0.596x^)	0.99
Volume (cm^3^)	Y = 184.92/(1 + 38.635 × 10^−0.63x^)	0.99

*Note*: The *p*-values of all the logistic regression dependent variables are less than 0.0001, indicating these models were significant at the 95% confidence interval.

**Table 2:** Changes in the exocarp thickness of “terung asam” fruit harvested at three different growth stages (*n* = 12).

Growth stage	Exocarp thickness (μm)
Stage 1	3.92 c^z^
Stage 2	62.39 b
Stage 3	79.04 a

*Notes*:

z Means followed by the same letter in the same column are not significantly different by Duncan’s multiple range test at *p* < 0.05.
